# 1-Year Rehospitalization and Mortality Rates in Geriatric Patients after Acute Hospitalization

**DOI:** 10.1093/geroni/igaa057.3351

**Published:** 2020-12-16

**Authors:** Sun Paek, Elena Volpi, Rachel Deer

**Affiliations:** The University of Texas Medical Branch at Galveston, Galveston, Texas, United States

## Abstract

Hospitalization for an acute illness often leads to accelerated sarcopenia, debility, and loss of independence in geriatric patients. These post-hospitalization effects can further deteriorate health and lead to rehospitalizations and sometimes death. To improve health outcomes in these patients, it is important to determine predictive factors for increased readmission and mortality rates. Data regarding readmission and mortality within 1 year post-discharge were collected from the PACE and GRAMS studies (NCT02203656, NCT02990533) conducted at UTMB Galveston (Jan 2014 - Mar 2019). Readmission and mortality rates were analyzed to find associations with total days hospitalized and total number of readmissions within 1 year, demographics (age, highest education level, BMI, percent fat, baseline SPPB score), and interventions (exercise with protein supplementation, exercise with placebo, protein supplementation, placebo supplementation, and testosterone injection). Of the 175 subjects, 63.4% were rehospitalized and 9.7% died within 1 year. We found that patients who were rehospitalized within a year of their initial hospitalization were over 4 times more likely to die that year than patients who were not rehospitalized. Patients who died within one year had twice the amount of hospitalization within the year and spent three times the number of days hospitalized. Of the interventions studied, a single testosterone injection at discharge from the index admission appeared the most effective to reduce rehospitalizations.

